# The quality of the fossil record across higher taxa: compositional fidelity of phyla and classes in benthic marine associations

**DOI:** 10.7717/peerj.15574

**Published:** 2023-07-11

**Authors:** Carrie Tyler, Michał Kowalewski

**Affiliations:** 1 Department of Geoscience, University of Nevada, Las Vegas, Nevada, United States of America; 2 Florida Museum of Natural History, University of Florida, Gainesville, Florida, United States of America

**Keywords:** Paleoecology, Conservation, Taphonomy

## Abstract

Although the fossil record preserves a wealth of historical data about past ecosystems, the current paradigm, which postulates that fossils provide faithful archives of ecological information, stems from research primarily focused on a single group of organisms known for their high fossilization potential: molluscs. Here, we quantify the fidelity of higher taxa (six phyla and 11 classes) by comparing live communities and sympatric dead remains (death assemblages) using comprehensive surveys of benthic marine invertebrates from coastal habitats in North Carolina (U.S.A). We found that although community composition differed between the two assemblages across phyla and classes, these differences were predictable with an overabundance of robust and more preservable groups. In addition, dead molluscs appear to be an excellent proxy for all taxa when tracking spatio-temporal patterns and shifts in community structure using a variety of ecological metrics, including measures of *α*, *γ*, and *β* diversity/evenness. This suggests that despite filters imposed by differential preservation of taxa and time-averaging, the fossil record is likely to be reliable with respect to relative comparisons of composition and diversity in shallow benthic marine paleocommunities. This is consistent with previous work indicating that shallow marine death assemblages can yield robust ecological estimates adequate for assessing the variability of ecosystems that existed under natural, pre-anthropogenic conditions.

## Introduction

Paleontological data serve as archives of past ecosystems, and can offer a long-term pre-industrial perspective that can enhance ecosystem management and restoration (Willis & Birks, 2006; Smith, Durham & Dietl, 2018; Tyler & Schneider, 2018; Kidwell & Tomašových, 2013). As records documenting the natural variability of ecosystems are largely absent, determining the extent to which paleontology can provide meaningful estimates of past ecological conditions is becoming increasingly important (Stempien, 2005; Willis & Birks, 2006; Knowlton & Jackson, 2008; Louys, Wilkinson & Bishop, 2012; Kidwell & Tomašových, 2013; Kowalewski et al., 2015; Smith, Durham & Dietl, 2018; Tyler & Schneider, 2018; Gallmetzer et al., 2019; Hyman et al., 2019; Albano et al., 2021). Direct comparisons between live communities and sympatric dead remains (*i.e.,* death assemblages) have been fruitful in evaluating the reliability of paleontological data when reconstructing past ecological conditions, such as community composition and diversity (also known as ecological fidelity). Live-dead comparisons suggest that molluscan death assemblages accurately record a wide range of ecological patterns (*e.g.*, see Kidwell, Bosence & Bosence, 1991a; Kidwell, 2001; Kidwell, 2002; Kidwell, 2007; Tomašových & Kidwell, 2009; Kidwell, 2013; Dietl et al., 2016; Tyler & Kowalewski, 2017; Pruden et al., 2021), and in some cases molluscs have been used in fidelity studies as surrogates for entire communities with promising results (Tyler & Kowalewski, 2014; Tyler & Kowalewski, 2017). However, while the compositional fidelity of species and genera has been widely studied for molluscs, live-dead comparisons of phyla and classes remained understudied, as noted by many authors (*e.g.*, Staff et al., 1986; Kidwell, Bosence & Bosence, 1991a; Behrensmeyer, Kidwell & Gastaldo, 2000; Lockwood & Chastant, 2006; Albano & Sabelli, 2011; Weber & Zuschin, 2013; García-Ramos et al., 2016; Bürkli & Wilson, 2017). Moreover, the few multi-taxic case studies known to us were all restricted to a small subset of higher taxa (Schopf, 1978; Staff & Powell, 1988; Martin et al., 1996; Kowalewski et al., 2003; Stempien, 2005; Tyler & Kowalewski, 2017).

Several factors can bias the death assemblage (DA) including but not limited to differential preservation, time-averaging, bioerosion, dissolution, transport, and the life-span of organisms. Greater richness and abundance of more durable taxa with more heavily biomineralized exoskeletons in DAs, such as molluscs, may arise due to differential preservation (Kidwell, 2001; Korpanty & Kelley, 2014). Differences in durability, bioerosion, and dissolution may also influence temporal resolution, with more durable taxa undergoing more substantial time-averaging (Kowalewski, 1997; Behrensmeyer, Kidwell & Gastaldo, 2000; Kowalewski et al., 2018, but see Nawrot et al., 2022). Organisms with more robust hard parts may take longer to dissolve or erode relative to those with fragile skeletons or predominantly soft-tissues, increasing time-averaging and creating differential time-mixing across taxa (Broecker & Clark, 2011; Mekik, 2014; Kowalewski et al., 2018, but see Nawrot et al., 2022). Although post-mortem transport most commonly leads to coarser spatial resolution in DAs (Kidwell & Tomašových, 2013), transport likely only affects a few individuals (Flessa, 1998), and is not expected to have a substantial impact on compositional fidelity in shallow marine soft-bottom habitats (Kidwell, Bosence & Bosence, 1991a; Nebelsick, 1992; Kidwell & Flessa, 1995; Miller et al., 1997; Kowalewski, Goodfriend & Flessa, 1998; Behrensmeyer, Kidwell & Gastaldo, 2000; Best, 2008; Kidwell, 2008; Albano & Sabelli, 2011; Weber & Zuschin, 2013; García-Ramos et al., 2016; Casebolt & Kowalewski, 2018; Hyman et al., 2019, but see Bürkli & Wilson, 2017). Little is known regarding the effect of life-span on fidelity. Organisms with short life-spans may be more commonly preserved, as they are more frequently added to DAs (Cadee, 1968; Vermeij & Herbert, 2004; Albano & Sabelli, 2011; Cronin et al., 2018). But it has also been argued that variation in life-span may have little effect on DA composition (Cummins et al., 1986) or the fidelity of bivalves in soft-bottom habitats (Kidwell & Rothfus, 2010).

Diversity is also known to vary between live and dead, and *α* and *γ* are typically elevated in mollusc DAs (Tomašových & Kidwell, 2009; García-Ramos et al., 2016), which often incorporate a greater proportion of the regional species pool, while *β*-diversity is often underestimated (Tomašových & Kidwell, 2009; Kidwell & Tomašových, 2013; Weber & Zuschin, 2013; García-Ramos et al., 2016; Tyler & Kowalewski, 2017; Hyman et al., 2019). Although evenness is likely to be inflated in DAs due to time-averaging, it may also be affected by differential time-averaging among groups (Fürsich & Aberhan, 1990; Kidwell, 2001; Olszewski & Kidwell, 2007; Kidwell & Tomašových, 2013; Weber & Zuschin, 2013; García-Ramos et al., 2016 but see Albano & Sabelli, 2011; Korpanty & Kelley, 2014).

In addition to identifying the limits of the utility of DAs and paleontological data in conservation, understanding the relative fidelity of multiple types of organisms is also necessary for the study of macro-evolutionary patterns in which the fossil record is often analyzed jointly across multiple phyla (*e.g.*, Stanley, 1979; Jablonski et al., 1983; Vermeij & Roopnarine, 2013). It remains unclear whether multi-taxic death and fossil assemblages provide meaningful estimates of diversity, relative abundance, and other aspects of local communities.

This study aims to quantify fidelity across multiple higher taxa simultaneously, including all phyla of macroscopic benthic organisms (six phyla and 11 classes in the study system analyzed here), and assess the extent to which death assemblages, and therefore the fossil record, provide accurate representations of live communities. Here we evaluate two critical components of ecological fidelity using live-dead comparisons in nearshore benthic marine invertebrate communities commonly employed in conservation paleobiology and paleoecology: composition and diversity.

## Methods

### Materials

To evaluate fidelity across multiple higher taxa simultaneously, concurrent live and dead samples were collected in a series of dredges over four field seasons (June 2011, November 2011, May of 2012, and April 2013) in Onslow Bay, North Carolina (U.S.A.). Dredging was conducted at 52 localities (Fig. S1), 16 of which were sampled in multiple years, resulting in a total of 220 samples collected from a variety of habitats, depths, and distances from shore (see Tyler & Kowalewski, 2018 and Supplemental Appendix for additional sampling details). Samples included invertebrates from six phyla (Annelida, Arthropoda, Brachiopoda, Cnidaria, Echinodermata, and Mollusca). All live macro-invertebrates that could be seen without the aid of a microscope were counted and identified to the lowest taxonomic level (typically species). Encrusting species (*e.g.*, bryozoans, barnacles, sponges) were excluded. Concurrent with live surveys, two bulk samples of dead material were collected at each locality (7.5 liters) and wet-sieved (mesh size 4.76 mm). As many organisms have multiple skeletal elements (*e.g.*, valves, plates, spines) that may disarticulate after death, each element was corrected by dividing element counts by the number of elements estimated to be contained within a single live individual (Tyler & Kowalewski, 2018). Skeletal fragments were excluded to prevent distortion of the DA composition which can result from inflating abundance estimates of species easily identifiable from small fragments (Kowalewski et al., 2003). Abundance data are available at https://github.com/tylercl/Multi-Taxic-Fidelity (DOI: 10.5281/zenodo.7871639).

### Compositional and diversity fidelity

Prior to all analyses small samples with fewer than 50 individuals were excluded (36 out of 52 sites were retained in the final analysis). As samples from DAs typically contain more individuals, sample standardization was performed using rarefaction, and samples were rarefied to the smallest of the two assemblages in any given live-dead pair. The fidelity of richness across and within phyla, classes, and species was estimated using Spearman’s and Pearson’s measures of correlations. For phyla and classes, taxon-focused estimates were derived for data pooled across all localities with live-dead pairs of points representing one regional estimate of species richness of a given higher taxon. For locality-focused estimates, species richness was estimated separately for each higher taxon and fidelity was measured as agreement between live and dead species richness within a given higher taxon across localities. Accelerated bias-corrected methods were used here to obtain improved estimates of confidence intervals obtained from bootstrap-derived distributions (*e.g.*, Chernick & LaBudde, 2011). Thus, to assess fidelity within phyla and classes, 95% confidence intervals were estimated using bootstrapping with an accelerated bias correction (1,000 iterations). Pearson’s correlations of richness, Spearman’s rank correlations of proportional rank abundance (calculated using the PaleoFidelity R package Kowalewski, 2023), and Bray-Curtis similarity were calculated using sample standardized data. The three measures of correlation/similarity tend to co-vary with one another but Pearson’s and Bray Curtis provide estimates weighted toward abundant taxa and are more sensitive to outliers. Spearman’s rank correlation weights all taxa equally regardless of their abundance.

To determine to what extent DAs provide meaningful estimates of diversity across multiple higher taxa simultaneously, the following diversity estimates were calculated (two sites with fewer than 20 individuals were removed to retain meaningful numbers of individuals across phyla): richness (S), Shannon’s H, Simpson’s D, and Pielou’s evenness (J). To assess the reliability of DAs in capturing diversity, three assemblages were compared: (1) the multi-taxic LA and the molluscan DA; (2) the multi-taxic LA and non-mollusc DA; and (3) the mollusc DA and the non-mollusc DA. Mean diversity between assemblage pairs was compared using a Kruskal-Wallis test with a Bonferroni correction using the rstatix package (Kassambara, 2021).

To assess whether compositional fidelity differed across localities due to skeletal robustness (*i.e.,* preservation biases), data were divided into two groups: molluscs, which have more durable skeletal components, and non-molluscs, with a larger proportion of species that are either soft-bodied or have less robust skeletal elements. This coarse grouping allows adequate retention of within-group sample sizes within localities. Similarity and rank-order agreement were then re-calculated for each subset separately using rarefied richness. Two sites with fewer than 20 individuals were excluded to retain meaningful numbers of samples and sites across phyla, leaving 50 sites.

To further assess compositional fidelity, the expected ‘perfect’ fidelity was estimated separately for each live-dead comparison. Fidelity estimates are highly sensitive to small sample sizes and unbalanced sampling. Unbalanced sampling is common when sampling the marine benthos (including this analysis) because live specimens tend to be far more rare relative to dead remains. It is therefore instructive to compare observed values of statistics of interest (*e.g.*, Spearman’s rank correlations) to those expected if the fidelity were perfect and departures from perfect concordance (*e.g.*, Spearman’s Rho = 1) was due to sampling alone. The estimates were obtained by randomization *via* reassignment of group membership by random resampling without replacement (*e.g.*, Kowalewski & Novack-Gottshall, 2010; Manly & Alberto, 2021). For a given live-dead comparison both live and dead specimens were pooled together to create a single species distribution and specimens were then assigned randomly to live and dead samples using their original sample sizes. Because this randomization samples a single pooled distribution, the fidelity should be perfect, and any departures (*e.g.*, Spearman’s Rho < 1) would reflect (and estimate) sampling effects (see also Kowalewski, 2023; PaleoFidelity R Package). Each randomization was based on 1,000 replicate samples.

Differences in multivariate dispersions between live and death assemblages were examined using a modified analysis of Homogeneity in Multivariate Dispersions (HMD; Tomasovych & Kidwell, 2011), which can be used to quantify the effects of premortem and postmortem variation. Analyses were conducted separately for the multi-taxic, mollusc, and non-mollusc assemblages. Two common dissimilarity measures were calculated, Jaccard dissimilarity which relies on presence/absence data, and Bray-Curtis, which utilizes species abundance data. Both measures were calculated using rarefied standardized data (six sites with fewer than 30 individuals were excluded, leaving 46 sites). Abundance was standardized by dividing the abundance of a given species by the total locality abundance. HMD was performed using a custom function in R (R Core Team, 2019) developed by Tomasovych & Kidwell (2011).

## Results

**Figure 1:**
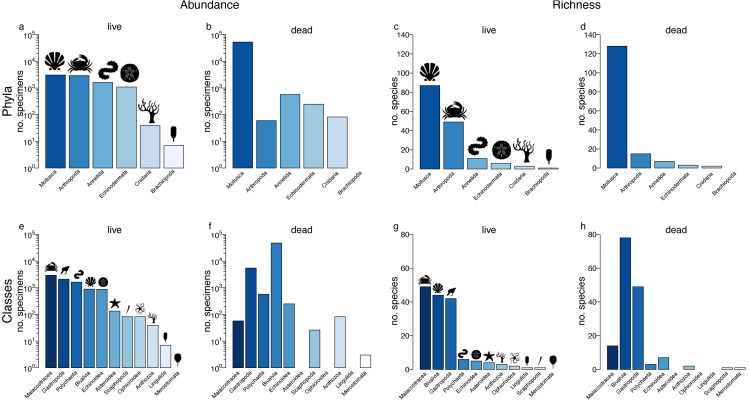
Relative abundance and richness across higher taxa. Specimen abundance and richness within phyla (A–D) and classes (E–H) for the live assemblage (A,C,E,G) and the death assemblage (B,D,F,H). Samples with n < 50 were removed, but data were not sample standardized.

Sympatric sampling of live communities and death assemblages *via* dredging in Onslow Bay, North Carolina (U.S.A.) yielded 221 samples from 52 sites. After removing 16 sites with small sample sizes (fewer than 50 individuals), the live assemblage (LA) totaled 8,798 individuals representing 157 species from six phyla and 11 classes, whereas the death assemblage (DA) totaled 54,116 individuals representing 155 species from five phyla and 10 classes (Fig. 1; Table 1; Table 2).

**Table 1:** Abundance and richness within phyla. The live-dead fidelity within phyla was low with the exception of molluscs (Pearson’s correlation). Missing values represent phyla with fewer than two species in either the live or dead. N = number of individuals, and samples with fewer than 50 specimens were removed (36 sites were retained), but data are not sample standardized. Pearson’s correlations (r), Spearman’s correlations (Rho), and associated *p*-values are shown for correlations between the live and death assemblages within phyla. For example, sites with a greater number of mollusc species in the live assemblage also had higher mollusc richness in the death assemblage (see Fig. S2B for the correlation among mollusc sites, with n < 20 removed).

Phylum	Live N	Dead N	Rho	*p*	r	*p*	Live Sp.	Dead Sp.	Rho	*p*	r	*p*
Mollusca	3,076	53,151	0.26	0.13	−0.03	0.88	87	128	0.44	0.002	0.40	0.01
Arthropoda	2,944	60	0.39	0.02	0.34	0.05	49	15	0.37	0.03	0.40	0.02
Annelida	1,640	574	−0.21	0.22	−0.03	0.87	6	3	0.06	0.72	0.24	0.16
Echinodermata	1,092	249	0.42	0.01	0.14	0.43	11	7	0.17	0.33	0.24	0.16
Cnidaria	39	82	−0.12	0.47	0.14	0.41	3	2	−0.27	0.11	−0.24	0.17
Brachiopoda	7	0	–	–	–	–	1	0	–	–	–	–
Total	8,798	54,116					157	155				

**Table 2:** Abundance and richness of classes. The live-dead fidelity within classes was low with the exception of mollusks (Spearman’s Rho and Pearson’s r correlations). The correlation of abundance within each class was only significant for malacostraceans. Richness was only significantly correlated within malacostraceans, echinoderms, and bivalves. Both Spearman’s and Pearson’s correlations produced consistent results. Missing values represent classes with fewer than two species in either the live or dead. N = number of individuals, and samples with fewer than 50 specimens were removed, but data were not sample standardized.

Class	Live N	Dead N	Rho	*p*	r	*p*	Live Sp.	Dead Sp.	Rho	*p*	r	*p*
Malacostracea	2,944	57	0.40	0.02	0.33	0.05	49	14	0.34	0.05	0.33	0.05
Gastropoda	2,103	5,465	0.38	0.15	−0.10	0.57	42	49	0.36	0.03	0.30	0.08
Polychaeta	1,640	574	−0.21	0.22	−0.03	0.87	6	3	0.06	0.72	0.24	0.16
Bivalvia	890	47,660	0.46	0.005	0.21	0.22	44	78	0.45	0.006	0.45	0.006
Echinoidea	877	249	0.30	0.08	0.13	0.46	5	7	0.33	0.05	0.48	0.003
Ophioroidea	82	0	–	–	–	–	2	0	–	–	–	–
Asteroidea	133	0	–	–	–	–	4	0	–	–	–	–
Anthozoa	39	82	−0.12	0.47	0.14	0.41	3	2	−0.27	0.11	−0.24	0.17
Lingulida	7	0	–	–	–	–	1	0	–	–	–	–
Scaphopoda	83	26	–	–	–	–	1	1	–	–	–	–
Merostomata	0	3	–	–	–	–	0	1	–	–	–	–
Total	8,798	54,116					157	155				

### Diversity fidelity

At the coarsest, regional scale, a single live-data comparison can be carried out for higher taxa by pooling data across all localities. In terms of agreement in relative abundance of phyla, mollusc specimens dominated both the LA and DA (Figs. 1A–1B). With the exception of arthropods, rank-order abundance in the DA remained consistent with the LA. The live-dead agreement in abundance was lower at the class level, mostly reflecting discordances in bivalves (most abundant in the DA but ranked fourth in the LA) and arthropods (most abundant in the LA but ranked sixth in the LA). Nevertheless, four out of the five classes that were most abundant in the DA were also among the top five in the LA. Moreover, gastropods and polychaetes were ranked second and third in terms of specimen abundance in both the DA and LA samples (Figs. 1E–1F).

**Figure 2:**
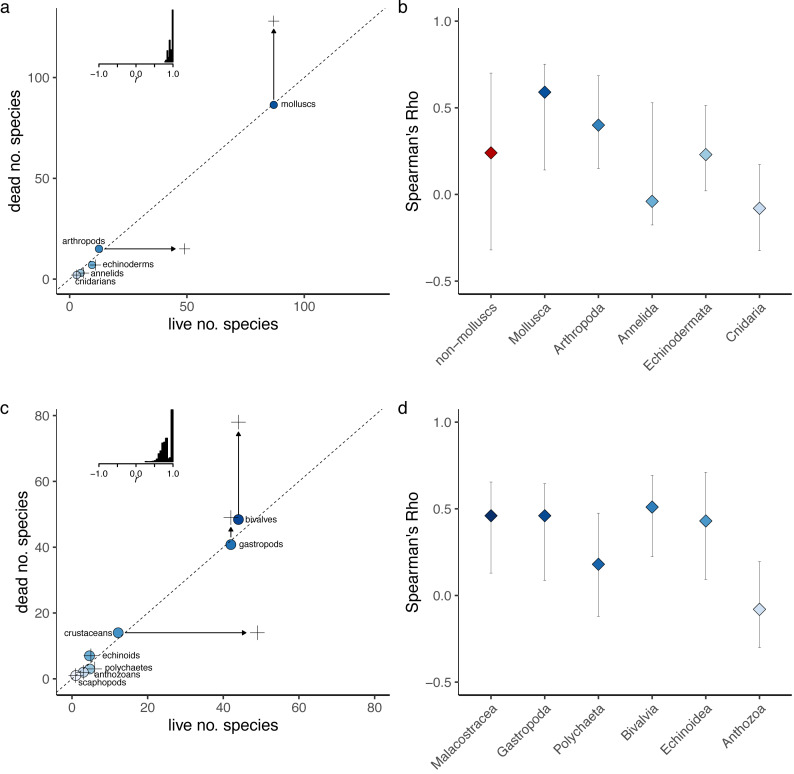
Live-dead agreement. The number of species within phyla (A) and classes (C) indicate predictable live-dead discordance. Filled circles indicate live richness and crosses dead richness. Arrows show the change in richness between the live assemblage and the death assemblage for higher taxa, and the dashed line denotes perfect fidelity. The fidelity of richness was also assessed within phyla (B) and classes (D) using Spearman’s correlations, with 95% confidence intervals calculated using an accelerated bootstrapped correction. Classes with fewer than three species were excluded. Samples with less than 20 individuals were removed, and samples were standardized. The Pearson’s correlations are provided in Fig. S5.

In terms of agreement in species diversity, molluscs were the most diverse phylum in both the LA and DA assemblages (Figs. 1C–1D; Table 1). The non-standardized species richness of phyla was positively correlated between the LA and DA assemblages (Spearman’s Rho = 1, *p* = 0.003; Pearson’s *r* = 0.91, *p* = 0.01; Fig. 2A; Table S1), with molluscs dominating both assemblages. In fact, the relationship was monotonic with a perfect live-dead agreement in rank abundance of the five phyla. The live-dead agreement in non-standardized species richness was also high when comparing classes, with the diversity of classes in the DA being a good predictor of diversity of classes in the LA (Pearson’s *r* = 0.79, *p* = 0.004; Spearman’s Rho = 0.79, *p* = 0.004; Fig. 2C; Table S1). As with molluscs, the bivalves and gastropods were the most diverse classes in the DA.

Phyla with high abundance in the LA also had high richness (Pearson’s *r* = 0.96, *p* = 0.003; Spearman’s Rho = 0.96, *p* = 0.0005), as did classes (Pearson’s *r* = 0.97, *p* < 0.001; Spearman’s Rho = 0.94, *p* < 0.001). This relationship was similarly strong in the DA for both phyla (Pearson’s *r* = 0.99, *p* < 0.001; Spearman’s Rho = 0.75, *p* = 0.05) and classes (Pearson’s *r* = 0.93, *p* < 0.001; Spearman’s Rho = 0.95, *p* < 0.001).

At the locality level, the correlation between the rarefied number of species in the multi-taxic LA and DA was strong and significant (Rho = 0.71, *p* < 0.001, 50 sites; Figs. S2A–S2B). Similarly, when assemblages were constrained to molluscs, locality level richness in mollusc LAs was strongly correlated with richness in sympatric mollusc DAs (Rho = 0.56, *p* < 0.001, 36 sites; Figs. S2C–S2D). The relationship was weaker and non-significant for non-molluscs (Rho = 0.02, *p* = 0.21, 17 sites; Figs. S2E–S2F). However, this could reflect the small number of samples in the non-mollusc assemblage (17 sites). The mollusc DAs were less informative as a proxy for site-level richness in the multi-taxic LA (*r* = 0.57, *p* = 0.02, 36 sites; Figs. S2G–S2H). When locality-level estimates of sample-standardized alpha diversity were obtained separately for each phylum (Fig. 2B), the correlation estimates were highest for molluscs and arthropods, but slightly lower than the multi-taxic correlation estimates reported above (Fig. 2B). The live-dead correlation was low for all other phyla (Fig. 2B). Similar results were obtained for locality-level sample standardized estimates of alpha diversity for classes, with highest positive correlations obtained for bivalves and echinoids (Fig. 2C). Non-sample standardized locality-level estimates of alpha diversity within phyla and classes (Tables 1 and 2) resulted in similarly strong correlations between live and dead molluscs and bivalves, but also produced elevated correlation values for arthropods, crustaceans, and echinoids. Results were comparable regardless of which correlation method was used (Spearman *versus* Pearson).

### Compositional Fidelity

**Figure 3:**
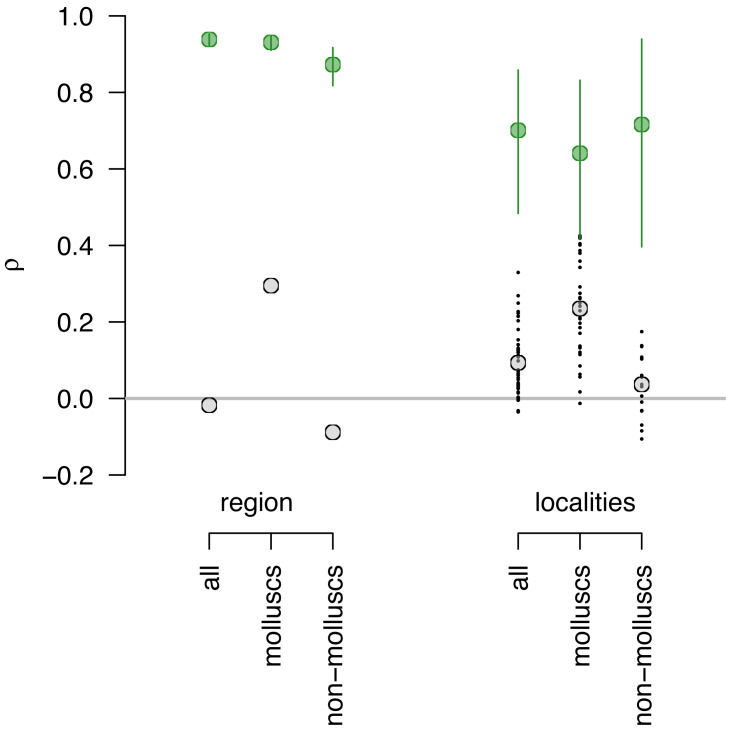
Assessing sampling effects using perfect fidelity comparisons. Sampling effects are relatively minor and do not vary notably across datasets. If fidelity were perfect (modeled values shown in green), estimates of Spearman’s Rho for regional data are expected to be around 0.9 for all three datasets (multi-taxic, molluscs only and non-molluscs) and cannot account for the very low Spearman’s Rho observed or differences in Rho values between molluscs and the other two datasets (observed values shown in gray). Similarly, at the locality level, the expected Rho values for perfect fidelity range between 0.6 and 0.8 and cannot account for differences between mollusks and non-molluscs or low correlation values.

At the regional scale with data pooled across all localities, compositional agreement for multi-taxi data is low (Fig. 3; Fig. S3A) regardless of the measure used (Spearman’s Rho = −0.01; Pearson’s *r* = 0.002; Bray-Curtis similarity = 0.09). The fidelity was slightly higher for molluscs (Fig. 3; Fig. S3A) when using rank correlation (Spearman’s Rho = 0.29) but comparably low using other measures (Pearson’s *r* = 0.02; Bray-Curtis similarity = 0.08). Similarly to multi-taxic data, the compositional fidelity is low for non-molluscs (Fig. 3; Fig. S3A) with rank correlation Spearman’s Rho of −0.002. However, other measures are somewhat elevated for non-molluscs (Pearson’s *r* = 0.31; Bray-Curtis similarity = 0.10). Nevertheless, regardless of grouping and measures used, the LA and DA samples are mostly or entirely discordant in terms of their species composition. Similar patterns are observed when compositional fidelity is analyzed at the locality level, with Spearman’s rank correlation (Fig. 3; Fig. S3A) notably higher for molluscs when compared to non-mollusc and multi-taxic data.

Because measures of correlation and similarity can be affected by sample size and unequal sample sizes, and both those issues affect the data analyzed here, it is useful to assess whether sampling biases can explain the observed low compositional fidelity. However, the resampling model of perfect fidelity indicates that the sampling effects are relatively minor and do not vary notably across datasets (Fig. 3). That is, if fidelity were perfect, the estimates of Spearman’s Rho for regional data are expected to be around 0.9 for all three datasets (Fig. 3) and cannot account for the very low Spearman’s Rho observed for all datasets or differences in observed Rho values between molluscs and the other two datasets. Similarly, at the locality level, the expected Rho values for perfect fidelity range between 0.6 and 0.8 and cannot account for differences between mollusks and non-molluscs or low correlation values.

**Figure 4:**
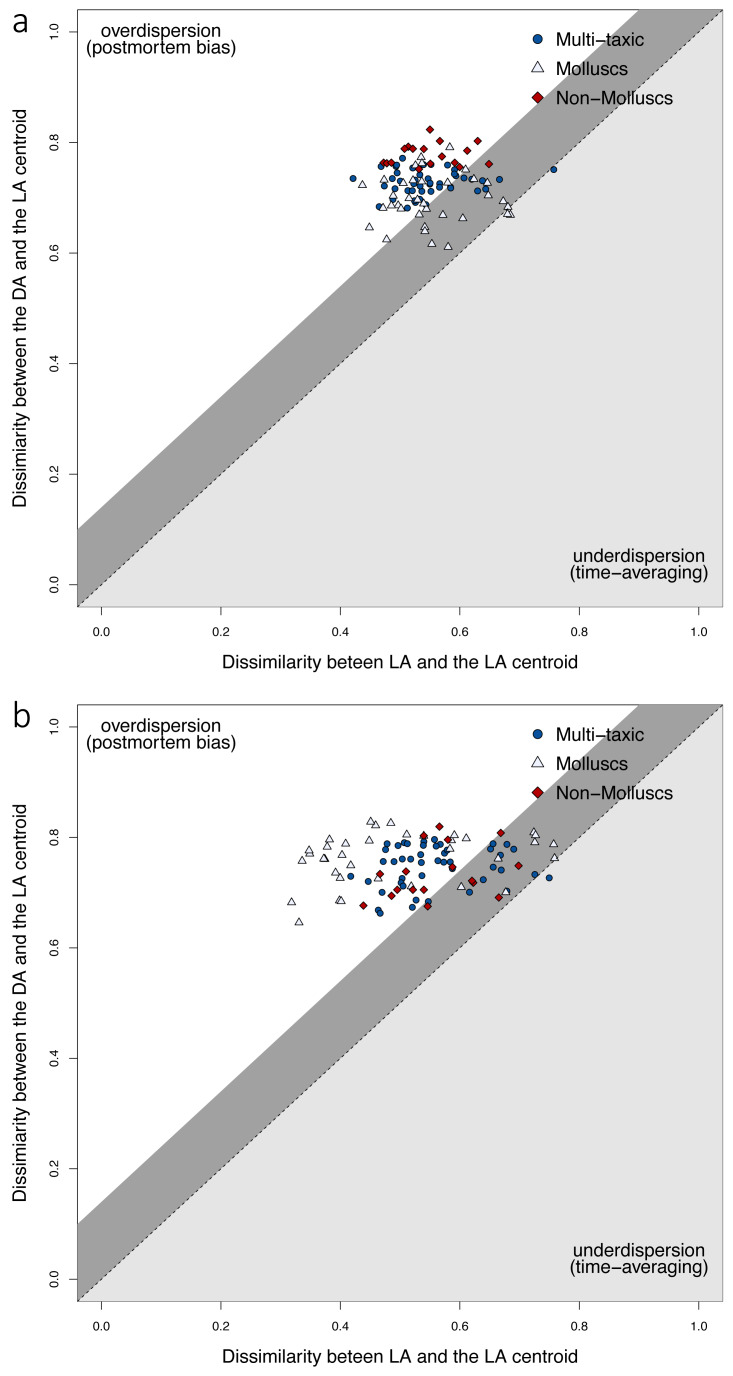
Live-dead differences in multivariate dispersions. Premortem variation among LAs (*x*-axis) relative to total live-dead variation (*y*-axis) using Jaccard (A) and Bray-Curtis (B) dissimilarities. Sites with fewer than 30 specimens were removed. Dissimilarity between the live and death assemblages at each site are shown for the full multi-taxic assemblage, and the mollusc and non-mollusc subsets. The shaded dark gray region above the dashed line of good agreement defines the magnitude of change in composition caused by within-habitat time-averaging of live assemblages, and live-dead dissimilarity for sites that fall in that region can be explained entirely by within habitat time-averaging (Tomasovych & Kidwell, 2011). In the upper white triangle, variation among death assemblages and the live assemblage centroid is greater than the variation among live assemblages and their centroid (*i.e.,* overdispersion). For the multi-taxic and mollusc assemblages, several sites fall on or somewhat above the expected line of good agreement due to within-habitat time-averaging (Tomasovych & Kidwell, 2011) using either dissimilarity metric. However, the non-mollusc assemblage did not have any sites in this plot region when using Jaccard dissimilarity. All three assemblages had some sites that were over-dispersed, with death assemblages that were affected by postmortem processes.

At the locality level, tests for Homogeneity in Multivariate Dispersions (HMD) indicate that DAs do not occupy the same region of the multivariate space as LAs, and are thus overdispersed relative to the centroid of LAs (Fig. 4). This pattern is observed consistently for the multi-taxic assemblage, mollusc and non-mollusc assemblages. However, multivariate dispersion was less pronounced in mollusc assemblages relative to the multi-taxic and non-mollusc assemblages when using Jaccard dissimilarity. All three DAs were significantly over-dispersed, however, the magnitude of overdispersion was somewhat low (0.15−0.27), and total live-dead variation was larger than premortem variation using either presence-absence or proportional abundance (Table S2). The proportion of total variation explained by premortem variation was similar in all three assemblages using both presence-absence (Jaccard) and standardized relative abundance (Bray-Curtis; Table S2). However, the magnitude of the total live-dead variation explained by the premortem component was marginally larger for mollusc assemblages relative to the multi-taxic assemblage when using Jaccard dissimilarity (0.63 *vs.* 0.57) and smaller when using Bray-Curtis (0.45 *vs.* 0.57; Table S2). Conversely, the non-mollusc assemblage was lower using Jaccard (0.47) and marginally higher using Bray-Curtis (0.61).

## Discussion

Consistent with previous studies, molluscs and other heavily biomineralized taxa were, not surprisingly, over-represented in the DA (Cummins et al., 1986; Kidwell, 2001; Kidwell, 2002; Kidwell, 2007; Olszewski & Kidwell, 2007; Tomašových & Kidwell, 2009; Bürkli & Wilson, 2017, but see Albano & Sabelli, 2011). Groups dominated by soft-parts/low-biomineralization such as arthropods, were typically represented in the DA by fewer individuals. Although at the phylum and class level the fidelity of the relative proportions of taxa were high (*e.g.*, rank-order abundance), this relationship broke down with increasing taxonomic resolution, and species-level live-dead compositional agreement within phyla and classes was low. The strong positive relationship between abundance and diversity across phyla and classes in the LA was similarly strong in the DA, supporting the idea that the number of species may increase with the number of individuals (*e.g.*, Bambach, 1993; Huntley & Kowalewski, 2007), which here holds true across taxonomic groups. That is phyla and classes with high abundance also had high diversity, a relationship that was preserved in the DA. The DA also had high fidelity in terms of diversity measures (Fig. S6), particularly within the most durable phylum: molluscs. Differences in quantitative measures of community structure and diversity were consistent with previous studies of molluscs, *i.e.,* depressed *β*, elevated *α* and evenness (Kidwell, 2002; Olszewski & Kidwell, 2007; Tomašových & Kidwell, 2009; Tyler & Kowalewski, 2017). Molluscs outperformed other groups providing more reliable measures, validating their use to characterize spatio-temporal patterns representative of entire communities, and the use of molluscs as surrogates for the community.

At the locality level, the fidelity of richness was fair across phyla and classes; within phyla, molluscs were significantly and moderately correlated (Table S1), and within groups at the class level, arthropods, gastropods, molluscs, and echinoids were significantly and moderately correlated (Table S2). Inflated DA richness and abundance is likely due to time-averaging (Fürsich & Aberhan, 1990; Kidwell, 2001; Albano & Sabelli, 2011; Kidwell & Tomašových, 2013). However, uniform effects of time-averaging across taxa cannot be assumed. Brachiopods and corals appear to experience extensive age mixing comparable to that of molluscs, whereas echinoids may undergo sub-decadal time averaging (Edinger et al., 2007; Krause et al., 2010; Kowalewski et al., 2018, but see Nawrot et al., 2022). The HMD analyses suggest that live-dead compositional differences were largely due to postmortem effects such as differential preservation, turnover, or human activities (Tomasovych & Kidwell, 2011; Kidwell, 2013). Notably, these patterns were consistent across all three data types, and both the molluscan and non-molluscan assemblages produced comparable dissimilarities to the multi-taxic assemblage. Thus, regardless of the source of bias, their effects were comparable for the multi-taxic assemblage, molluscs, and non-molluscs. In other words, postmortem processes resulted in proportionally similar directional effects across phyla, and molluscs captured the direction of compositional changes observed in the multi-taxic assemblage. Significantly, postmortem bias was somewhat reduced in molluscs using Jaccard dissimilarity, however, live-dead discordance was more pronounced using standardized relative abundance data (Bray-Curtis). Molluscs therefore provided a less biased record than multi-taxic or non-molluscan assemblages when using presence-absence data. The overall strong performance of molluscs is particularly important for conservation and monitoring, as targeted data collection efforts could be constrained to molluscs as surrogates for the whole fauna when assessing composition and diversity, expending fewer resources. For example, molluscs have been shown to perform well as surrogates for whole-fauna when using benthic indices for remediation (Dietl et al., 2016; Tyler & Kowalewski, 2017; Pruden et al., 2021; Kokesh et al., 2022).

Compositional differences between the LA and DA were unlikely to be the result of out-of-habitat transport or under-sampling of LAs. As samples were largely collected on a shallow shelf, which are typically unaffected by postmortem transport of species from other habitats, it is unlikely that transport significantly altered DAs. Similarly, out of habitat transport is uncommon on wide shelves sediment-starved (Kidwell, Bosence & Bosence, 1991b; Kidwell, 2013; Tyler & Kowalewski, 2017; Bhattacherjee et al., 2021). At each locality a minimum of three live and three dead samples were collected, and the number of samples ranged from 3–8 with a mean of four samples per locality. Sampling was conducted in multiple field seasons and years, and 13 localities were sampled twice (*i.e.,* in two different field seasons), and three localities were sampled three times (*i.e.,* in three different field seasons). Rarefaction curves of the complete data with all 52 sites suggest that overall sampling was adequate in both the LA and DA, with slopes that indicate that both the LA and DA were approaching the asymptote, suggesting that additional sampling effort yield would be low in regard to species (Fig. S4). As samples were largely collected on a shallow shelf, which are typically unaffected by postmortem transport of species from other habitats, it is unlikely that transport significantly altered DAs.

## Conclusions

Overall, agreement among phyla, classes and species was high, *e.g.*, phyla with higher richness in the LA had correspondingly high richness in the DA. This is particularly promising for comparisons of benthic shallow shelf assemblages in the fossil record, as numerous macro-evolutionary hypotheses rely on multi-taxic community data, and assume that relative proportions of richness across phyla, classes, or species are faithfully preserved. While the bias introduced by differences in the abundance and richness across phyla are commonly perceived as detrimental, our findings suggest that although incomplete, DAs may nevertheless provide a reasonable approximation of relative differences in community structure. The results are also consistent with other studies which find that molluscs are reliable surrogates for relative comparisons of composition and diversity in modern environments and in the fossil record (*e.g.*, Smith, 2005; Bevilacqua et al., 2009; Tyler & Kowalewski, 2017; Kokesh et al., 2022).

## Supplemental Information

10.7717/peerj.15574/supp-1 Supplemental Information 1 Supplemental Document

10.7717/peerj.15574/supp-2 Supplemental Information 2 Map of study area Points indicate locations of dredge samples. Inset box in the top left corner shows the study area relative to the state of North Carolina. Additional sampling information including GPS coordinates for each locality and sample dates can be found in Tyler & Kowalewski (2017) and Tyler & Kowalewski (2018).

10.7717/peerj.15574/supp-3 Supplemental Information 3 Correlation of richness among sites Samples with less than 20 individuals were removed leaving 50 sites, and richness was rarefied to the smallest sample size (either the live assemblage or the death assemblage). Sites (points) with high richness in the multi-taxic live assemblage (A–B) had correspondingly high richness in the death assemblage. When only molluscs were included (C–D), sites with a greater number of mollusc species in the live assemblage (LA) also had higher mollusc richness in the death assemblage (DA). Similarly, sites with high live assemblage non-mollusc richness had correspondingly high death assemblage non-mollusc richness (E–F), although this relationship was moderate, and not significant. Richness in mollusc death assemblages were also an excellent proxy for multi-taxic live assemblages (G–H), and sites with high richness in the multi-taxic live assemblage had correspondingly high richness in the mollusc death assemblage. The first column shows Spearman’s correlations (Rho), and the second column shows Pearson’s correlations (r).

10.7717/peerj.15574/supp-4 Supplemental Information 4 Live-dead proportional rank abundance of the 15 most abundant taxa in (A) the multi-taxic assemblage, (B) molluscs only, and (C) non-molluscs Taxa unique to either the LA or DA are shown in red, while taxa present in both the LA and DA are shown in green. Samples were standardized as above, and those with fewer than 20 specimens were removed.

10.7717/peerj.15574/supp-5 Supplemental Information 5 Rarefied richness per sampled site The accumulation of species for the full LA and DA (all 52 localities). Vertical bars represent the 95% confidence intervals. The curves were broadly congruent, with moderate offsets in the number of species between assemblages. Although the LA did not reach the asymptote, indicating moderate under-sampling, the slope suggests that the LA was nevertheless close to fully sampled and was approaching the asymptote. The slope of the DA suggests that the DA was somewhat more comprehensively sampled than the LA. Differences between the LA and DA are thus unlikely to be due to sampling incompleteness.

10.7717/peerj.15574/supp-6 Supplemental Information 6 Live-dead agreement The number of species within phyla (A) and classes (C) indicate predictable live-dead discordance. Filled circles indicate live richness and crosses dead richness. Arrows show the change in richness between the LA and DA for higher taxa, and the dashed line denotes perfect fidelity. The fidelity of richness was also assessed within phyla (B) and classes (D) using Pearson’s correlations, with 95% confidence intervals calculated using an accelerated bootstrapped correction. Classes with fewer than three species were excluded. Samples with less than 20 individuals were removed, and samples were standardized.

10.7717/peerj.15574/supp-7 Supplemental Information 7 Fidelity of diversity measures Sample standardized comparisons of Richness (S) (A), Shannon’s H (B), Simpson’s D (C), and Pielou’s J (D). For all measures of diversity, the mollusc DA does not differ significantly from the multi-taxic LA (Table S3). Mollusc death assemblages thus serve as reliable records of diversity in multi-taxic live assemblages. Samples with fewer than 20 individuals were removed.

## Additional Information and Declarations

### Data Availability

The following information was supplied regarding data availability: The data are available at GitHub and Zenodo: https://github.com/tylercl/Multi-Taxic-Fidelity
Carrie Tyler. (2023). tylercl/Multi-Taxic-Fidelity: Data for Tyler and Kowalewski2023 (Tyler&Kowalewski2023). Zenodo. https://doi.org/10.5281/zenodo.7871639.
