# Supplementary material for: The quality of the fossil record across higher taxa: compositional fidelity of phyla and classes in benthic marine associations
Source: PeerJ. 2023 Jul 11;11:e15574. doi: 10.7717/peerj.15574 (PMC10348303; doi:10.7717/peerj.15574)
Supplement: Supplemental Information 1 [file peerj-11-15574-s001.pdf]

Supplemental Material for: The quality of the fossil record across higher taxa: compositional fidelity of phyla and classes in marine benthic associations

METHODS

Live assemblages (LA) and death assemblages (DA) were sampled via dredging in Onslow Bay, North Carolina (U.S.A.) yielded 221 samples from 52 sites (Fig. 1). 16 Sites with small sample sizes ($n < 50$) were removed, results reported here are constrained to the resulting 36 localities. At each locality, a minimum of three samples were collected utilizing a benthic sled, a dredge basket, and a van Veen grab. The sled trawling duration at each locality was 5 minutes, and the basket trawling duration was 10 minutes. The benthic sled was lined with 1 mm wire mesh to ensure representative sampling of smaller species and juveniles. The use of multiple types of dredge equipment ensured that live infaunal organisms were adequately represented (27% of live species surveyed and 36% of live individuals were infaunal). All DA samples were wet-sieved (4.76 mm), air dried, and macro-invertebrates were identified to the lowest taxonomic level (typically species). Seasonal variation in faunal composition was assumed to be negligible, based on previous reports from the same study area (Day et al., 1971). Nevertheless, repeat sampling of localities at different times of the year were carried out to further minimize seasonal variation in sample composition and reduce the magnitude to which richness and relative abundances in the living population may be underestimated and inflate live-dead discordance (Kidwell, 2001a,b). For each species in the DA, a unique conversion factor was applied to each skeletal component to estimate the number of individuals. Molluscan shell fragments were excluded so as not to distort the composition of the death assemblage based on taxonomic distinctness (Kowalewski et al., 2003). In the case of multi-elemental skeletons, including decapod and xiphosurid skeletal remains, components were classified into anatomic categories (e.g., chelae, legs, carapace, etc.), and identified and counted separately. While conversions for some organisms were straightforward, such as decapods (one individual has 2 chelae), the exact number of a particular component that makes up a single individual is not always clear. An individual sea urchin, for example, has an unknown number of spines. In such cases, a high conservative conversion fraction was used (e.g., urchin spines were multiplied by 0.001). Rarefied species accumulation curves were used to determine sampling completeness using the entire dataset (all 52 localities).

RESULTS

Phyla and classes with high abundance and richness in the LA had similarly high values in the DA (Table 1). This relationship was weaker across species. Fidelity was also compared between the full multi-taxic data set and two subsets, molluscs only largely consisting of skeletally robust species, and non-molluscs consisting of soft-bodied and less robust species. When comparing the proportional abundance of the 15 most abundant taxa after sample standardization, fidelity was low to moderate with few shared taxa (ρ of 0.072 for the multi-taxic data set, 0.43 for molluscs, and 0.058 for non-molluscs), but was highest in the mollusc subset of the assemblage (Fig. 3). This was consistent with the within phyla correlations discussed above, with molluscs yielding the highest compositional fidelity. Mollusc DAs also yielded comparable results to the complete multi-taxic LA for sample standardized richness (S), Shannon's H, Simpson's D, and Pielou's evenness (Fig. 6). Pairwise comparisons of each diversity metric between the multi-taxic LA, mollusc DA, and non-mollusc DA indicated that the mollusc DA did not differ from the multi-taxic LA for all four diversity measures (Table 3).

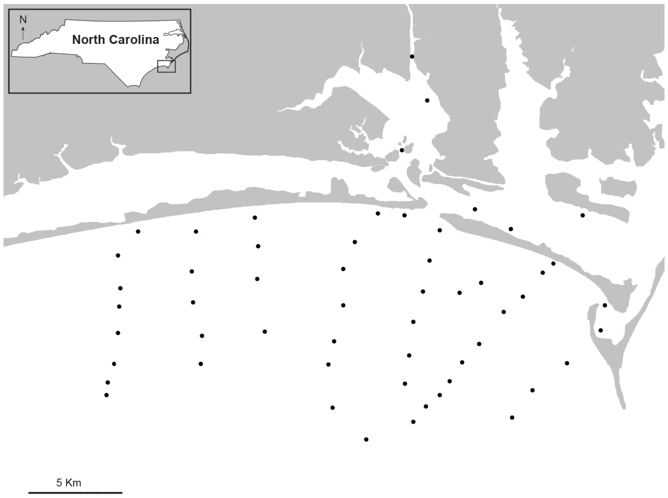

Figure 1. Map of study area. Points indicate locations of dredge samples. Inset box in the top left corner shows the study area relative to the state of North Carolina. Additional sampling information including GPS coordinates for each locality and sample dates can be found in (Tyler and Kowalewski, 2017, 2018).

REFERENCES

Day, J. H., Field, J. G., and Montgomery, M. P. (1971). The use of numerical methods to determine the distribution of the benthic fauna across the continental shelf of North Carolina. *Journal of Animal Ecology*, 40(1):93–125.

Kidwell, S. (2001a). Ecological fidelity of molluscan death assemblages. In Aller, J., Woodin, S., and Aller, R., editors, *Organism-Sediment Interactions*, pages 199–221. University of South Carolina Press, Columbia.

Kidwell, S. M. (2001b). Preservation of species abundance in marine death assemblages. *Science*, 294(5544):1091–1094.

Kowalewski, M., Carroll, M., Casazza, L., Gupta, N. S., Hannisdal, B., Hendy, A., Krause, R. A. J., LaBarbera, M., Lazo, D. G., Messina, C., Puchalski, S., Rothfus, T. A., Salgeback, J., Stempien, J. A., Terry, R. C., and Tomasovych, A. (2003). Quantitative fidelity of brachiopod-mollusk assemblages from modern subtidal environments of San Juan Islands, USA. *Journal of Taphonomy*, 1(1):43–66.

Tomašových, A. and Kidwell, S. M. (2009). Fidelity of variation in species composition and diversity partitioning by death assemblages: time-averaging transfers diversity from beta to alpha levels. *Paleobiology*, 35(1):94–118.

Tomašových, A. and Kidwell, S. M. (2011). Accounting for the effects of biological variability and temporal autocorrelation in assessing the preservation of species abundance. *Paleobiology*, 37(2):332–354.

Tyler, C. L. and Kowalewski, M. (2017). Surrogate taxa and fossils as reliable proxies of spatial biodiversity patterns in marine benthic communities. *Proceedings of the Royal Society B: Biology*, 284:20162839.

Tyler, C. L. and Kowalewski, M. (2018). Regional surveys of macrobenthic shelf invertebrate communities in Onslow Bay, North Carolina, U.S.A. *Scientific Data*, 5:180054.

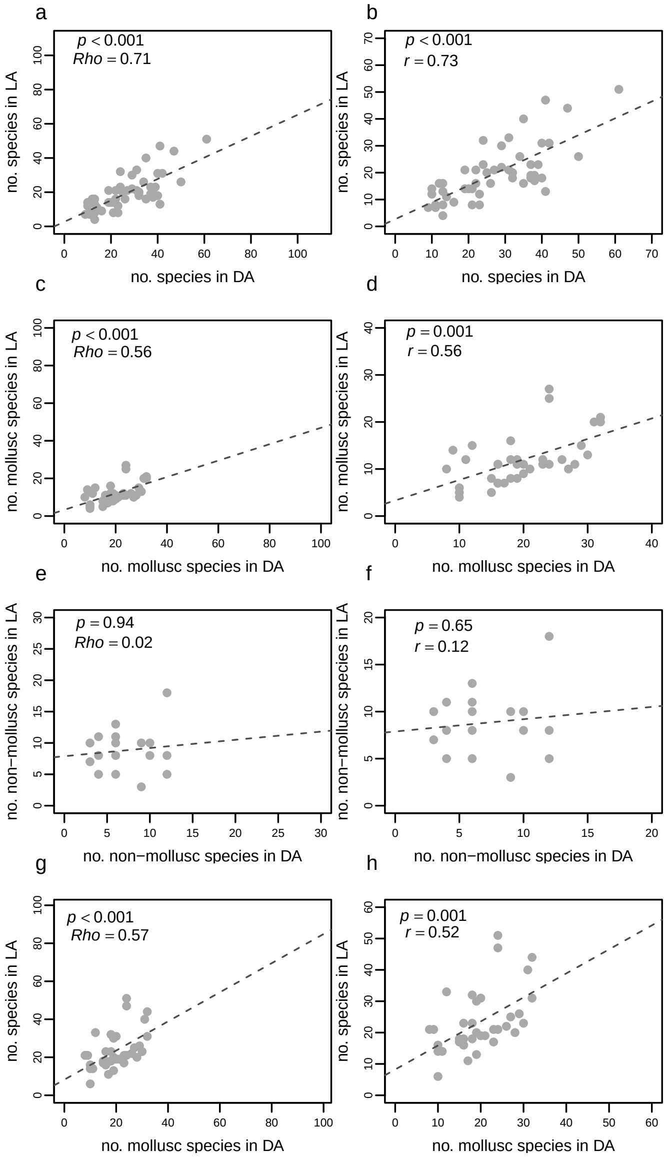

Figure 2. Correlation of richness among sites. Samples with less than 20 individuals were removed leaving 50 sites, and richness was rarefied to the smallest sample size (either the live assemblage or the death assemblage). Sites (points) with high richness in the multi-taxic live assemblage (a-b) had correspondingly high richness in the death assemblage. When only molluscs were included (c-d), sites with a greater number of mollusc species in the live assemblage (LA) also had higher mollusc richness in the death assemblage (DA). Similarly, sites with high live assemblage non-mollusc richness had correspondingly high death assemblage non-mollusc richness (e-f), although this relationship was moderate, and not significant. Richness in mollusc death assemblages were also an excellent proxy for multi-taxic live assemblages (g-h), and sites with high richness in the multi-taxic live assemblage had correspondingly high richness in the mollusc death assemblage. The first column shows Spearman's correlations (Rho), and the second column shows Pearson's correlations (r).

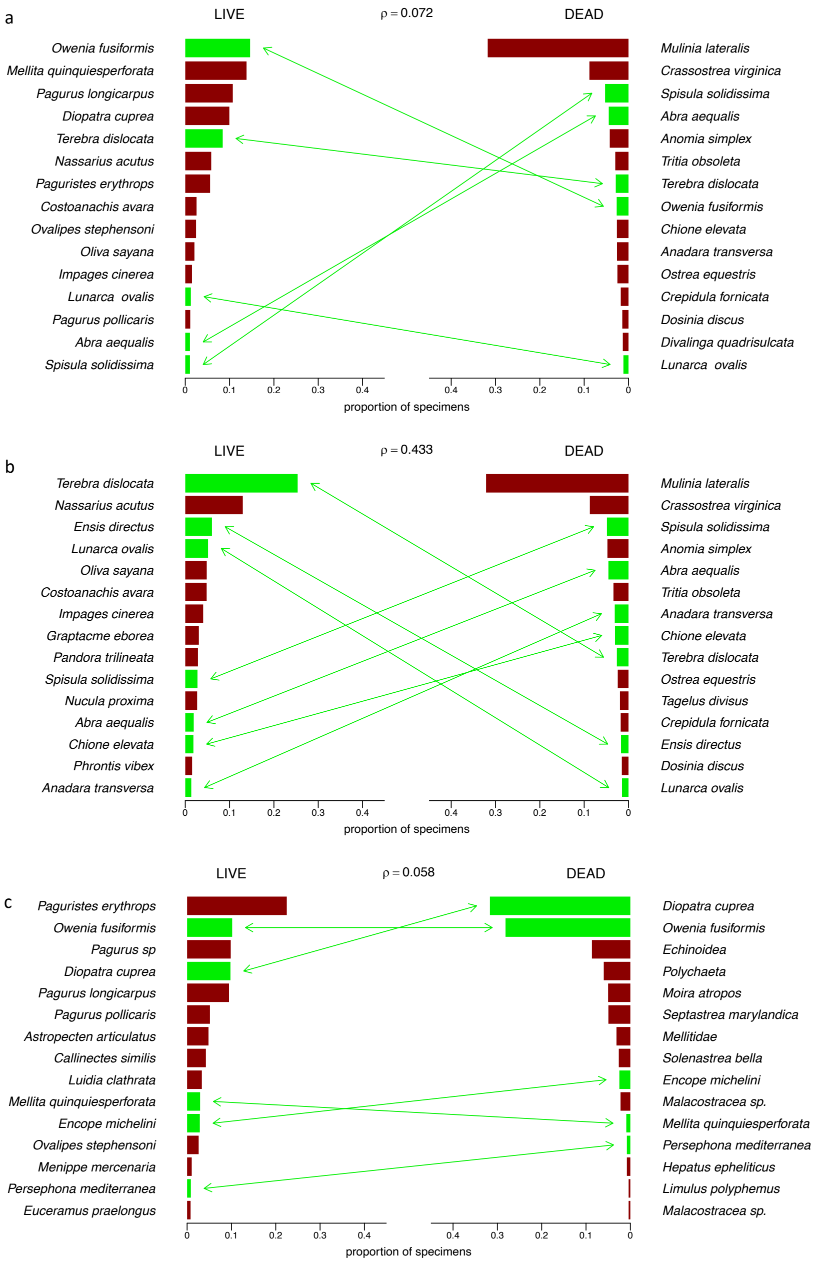

Figure 3. Live-dead proportional rank abundance. Live-dead proportional rank abundance of the 15 most abundant taxa in (a) the multi-taxic assemblage, (b) molluscs only, and (c) non-molluscs. Taxa unique to either the live or dead assemblage are shown in red, while taxa present in both the live and dead assemblage are shown in green. Samples were standardized as above, and those with fewer than 20 specimens were removed.

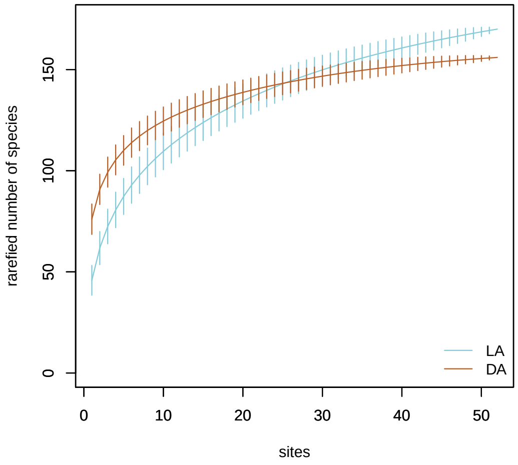

Figure 4. Rarefied richness per sampled site. The accumulation of species for the full live and dead assemblages (all 52 localities). Vertical bars represent the 95% confidence intervals. The curves were broadly congruent, with moderate offsets in the number of species between assemblages. Although the live assemblage (LA) did not reach the asymptote, indicating moderate under-sampling, the slope suggests that the live assemblage was nevertheless close to fully sampled and was approaching the asymptote. The slope suggests that the death assemblage (DA) was somewhat more comprehensively sampled than the live assemblage. Differences between the live and dead assemblages are thus unlikely to be due to sampling incompleteness.

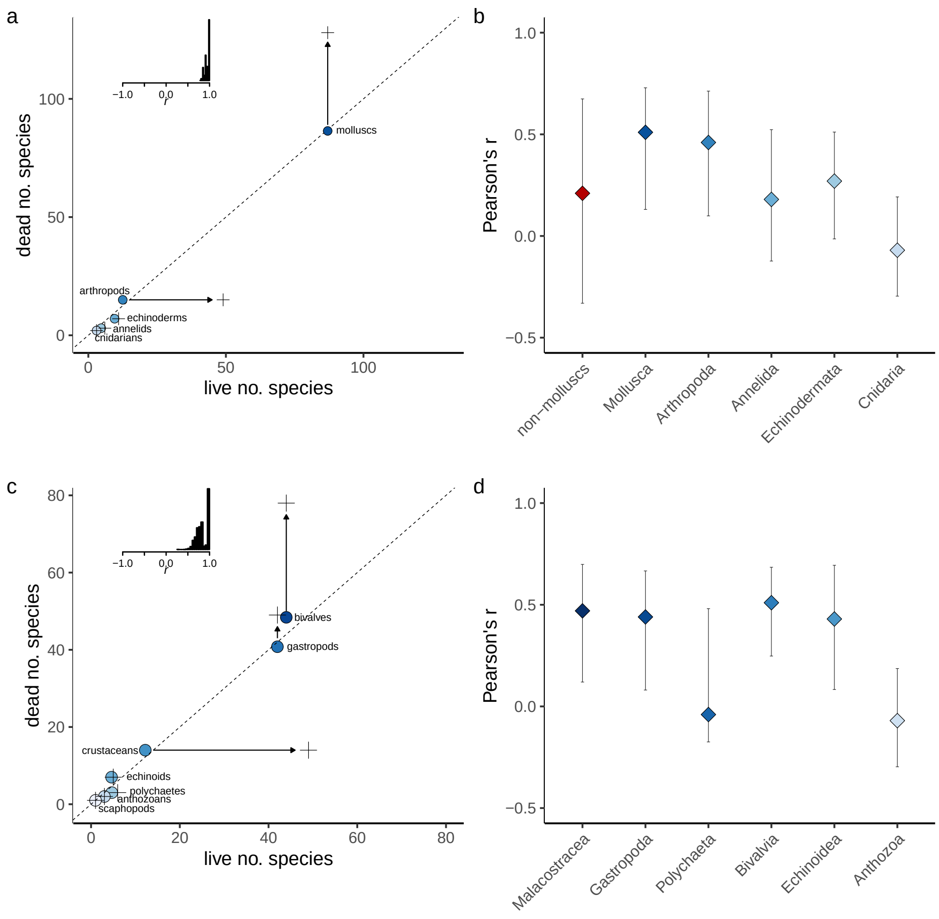

Figure 5. Live-dead agreement. The number of species within phyla (a) and classes (c) indicate predictable live-dead discordance. Filled circles indicate live richness and crosses dead richness. Arrows show the change in richness between the live and dead assemblages for higher taxa, and the dashed line denotes perfect fidelity. The fidelity of richness was also assessed within phyla (b) and classes (d) using Pearson's correlations, with 95% confidence intervals calculated using an accelerated bootstrapped correction. Classes with fewer than 3 species were excluded. Samples with less than 20 individuals were removed, and samples were standardized.

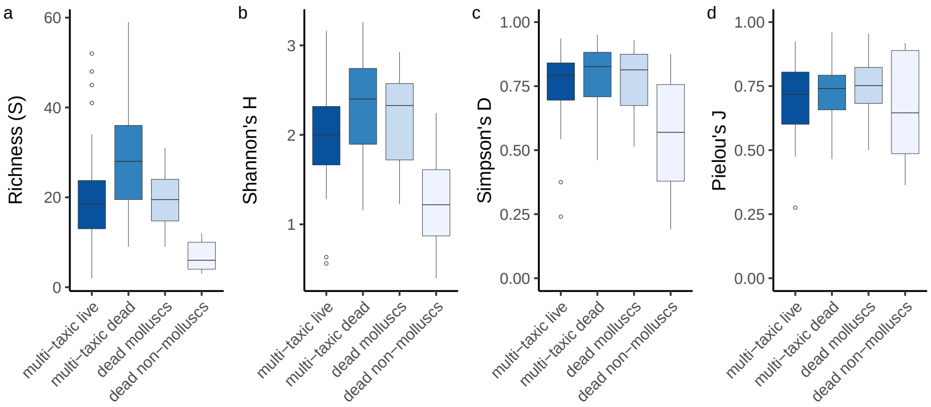

Figure 6. Fidelity of diversity measures. Sample standardized comparisons of Richness (S) (a), Shannon's H (b), Simpson's D (c), and Pielou's J (d). For all measures of diversity, the mollusc DA does not differ significantly from the multi-taxic LA (Table S3). Mollusc death assemblages thus serve as reliable records of diversity in multi-taxic live assemblages. Samples with fewer than 20 individuals were removed.

Table 1. Correlations between phyla, classes, and species. Both Spearman's and Pearson's correlations were performed to assess the live-dead fidelity of abundance and richness. Correlations of phyla and classes are based on the total taxa per phylum (df = 5) or class (df = 10). Correlations of species are comparisons of richness between localities.

	Phylum		Class		Species	
	Abundance	Richness	Abundance	Richness	Abundance	Richness
Rho	0.66	1	0.63	0.79	0.30	0.59
p	0.018	0.003	0.04	0.004	0.08	0.0002
r	0.59	0.91	0.82	0.79	0.11	0.56
p	0.22	0.01	0.81	0.004	0.52	0.0004

Table 2. Homogeneity in multivariate dispersions. Comparisons between the live (LA) and death assemblages (DA) for the multi-taxic assemblage and the mollusc and non-mollusc components. Total live-dead variation is the mean centroid of the live-dead distance, and premortem variation is the mean centroid of the live-live distance. P-values indicate the significance of tests of homogeneity in dispersions, and whether variation among death assemblages and the live assemblage centroids are larger (overdispersion), or smaller (underdispersion) relative to variation among live assemblage centroids. The proportion of total variation explained by premortem variation was calculated using sums of squared dissimilarities, and the ratio is shown (premortem variation/total live-dead variation) for each dissimilarity metric and assemblage, *sensu* (Tomašových and Kidwell, 2011, 2009).

	Multi-taxic Assemblage		Mollusc Assemblage		Non-mollusc Assemblage	
	Jaccard	Bray-Curtis	Jaccard	Bray-Curtis	Jaccard	Bray-Curtis
Proportion of total variation explained by premortem variation	0.57	0.57	0.63	0.45	0.47	0.61
Total live-dead variation	0.73	0.75	0.68	0.76	0.78	0.72
Premortem variation	0.55	0.56	0.54	0.50	0.54	0.56
Postmortem variation	0.18	0.19	0.15	0.27	0.25	0.16
p	< 0.001	< 0.001	< 0.001	< 0.001	< 0.001	< 0.001

Table 3. Pairwise comparisons between the multi-taxic live assemblage (LA), multi-taxic death assemblage (DA), mollusc death assemblage, and non-mollusc death assemblage for Richness (S), Shannon's H, Simpson's D. A Bonferroni correction was applied, and adjusted p-values are reported. The mollusc death assemblage does not differ from the multi-taxic live assemblage for all diversity measures.

	Statistic	adjusted *p*
**Richness (S)**		
Multi-taxic LA vs. multi-taxic DA	3.36	< 0.001
Multi-taxic LA vs. mollusc DA	0.2	1.00
Multi-taxic LA vs. non-mollusc DA	-4.7	< 0.001
Multi-taxic DA vs. mollusc DA	-2.8	< 0.001
Multi-taxic DA vs. non-mollusc DA	-7.0	< 0.001
Mollusc DA vs. non-mollusc DA	-4.6 < 0.001	
**Shannon's H**		
Multi-taxic LA vs. multi-taxic DA	3.2	0.009
Multi-taxic LA vs. mollusc DA	1.6	0.64
Multi-taxic LA vs. non-mollusc DA	-3.3	< 0.006
Multi-taxic DA vs. mollusc DA	-1.3	< 1.00
Multi-taxic DA vs. non-mollusc DA	-5.7	< 0.001
Mollusc DA vs. non-mollusc DA	-4.4 < 0.001	
**Simpson's D**		
Multi-taxic LA vs. multi-taxic DA	1.29	< 1.00
Multi-taxic LA vs. mollusc DA	0.61	1.00
Multi-taxic LA vs. non-mollusc DA	-2.9	< 0.02
Multi-taxic DA vs. mollusc DA	-0.6	< 1.0
Multi-taxic DA vs. non-mollusc DA	-3.8	< 0.001
Mollusc DA vs. non-mollusc DA	-3.2 < 0.008	
**Pielou's evenness (J)**		
Multi-taxic LA vs. multi-taxic DA	0.4	< 1.00
Multi-taxic LA vs. mollusc DA	1.2	1.00
Multi-taxic LA vs. non-mollusc DA	-0.8	< 1.00
Multi-taxic DA vs. mollusc DA	0.8	< 1.00
Multi-taxic DA vs. non-mollusc DA	-1.1	< 1.00
Mollusc DA vs. non-mollusc DA	-1.6 < 0.7	
