# Supplementary figures and images for: The quality of the fossil record across higher taxa: compositional fidelity of phyla and classes in benthic marine associations
Source: PeerJ. 2023 Jul 11;11:e15574. doi: 10.7717/peerj.15574 (PMC10348303; doi:10.7717/peerj.15574)

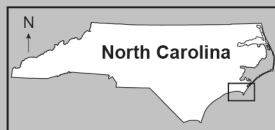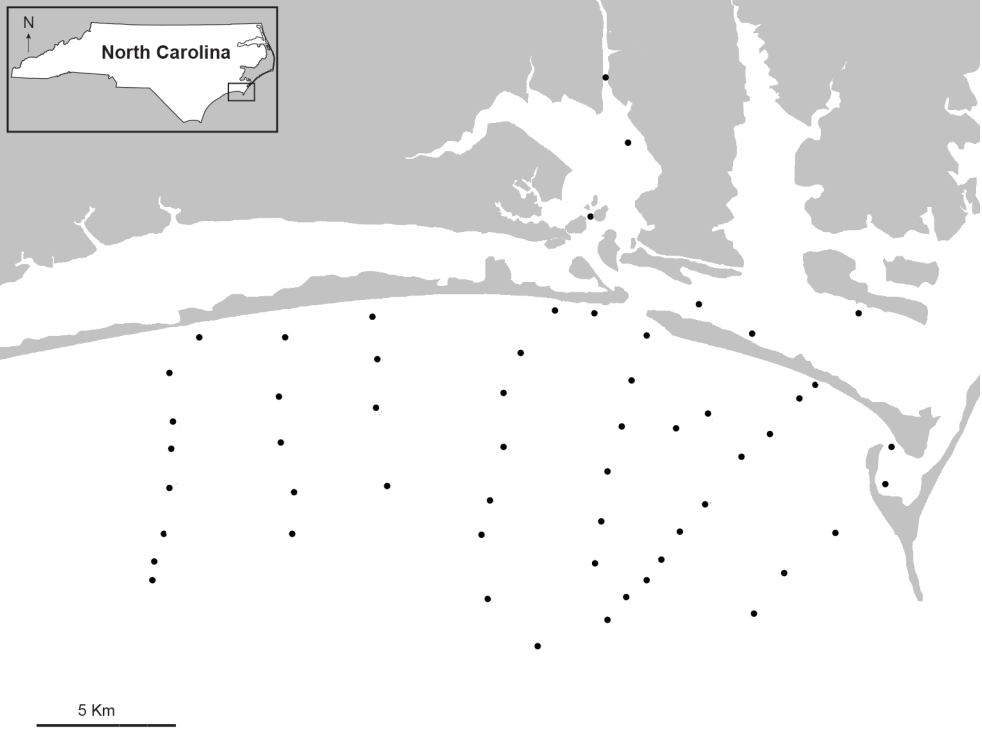

Supplement: Supplemental Information 2 — Points indicate locations of dredge samples. Inset box in the top left corner shows the study area relative to the state of North Carolina. Additional sampling information including GPS coordinates for each locality and sample dates can be found in Tyler & Kowalewski (2017) and Tyler & Kowalewski (2018). [file peerj-11-15574-s002.pdf]

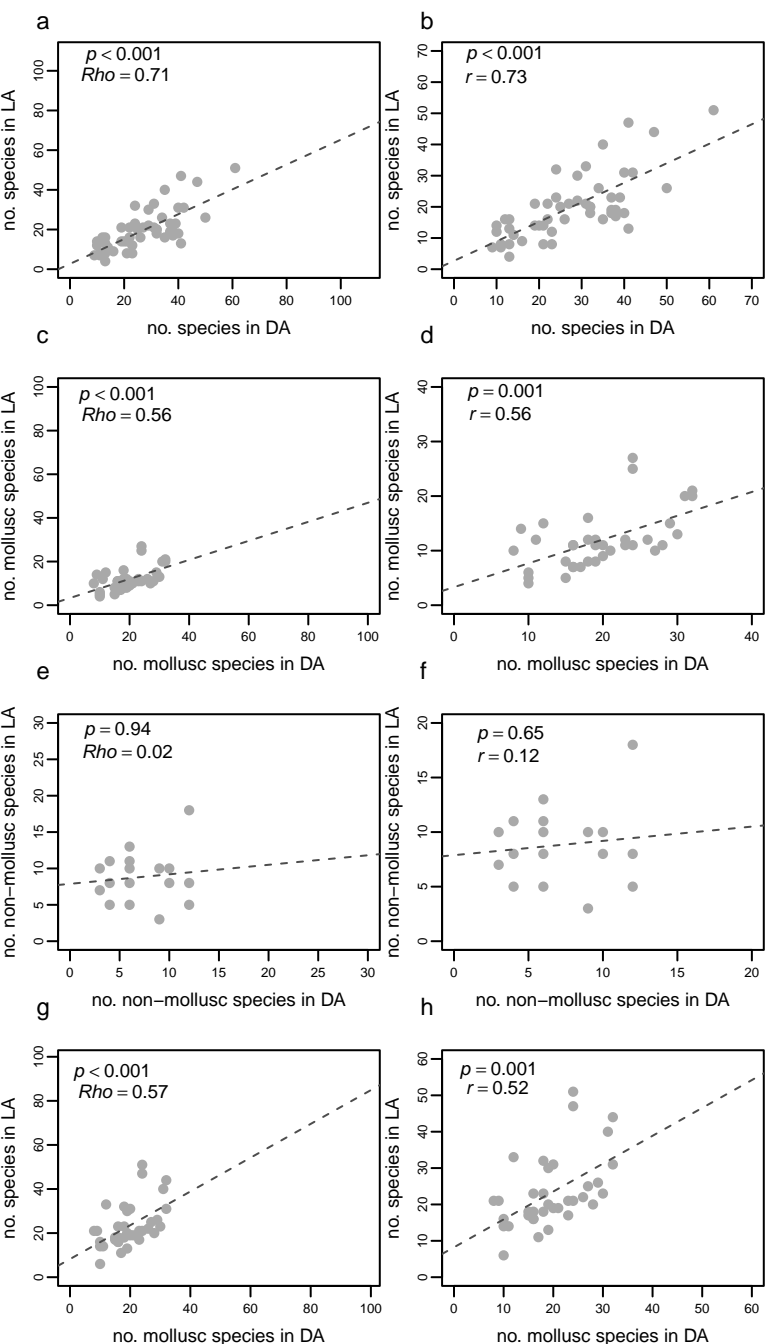

Supplement: Supplemental Information 3 — Samples with less than 20 individuals were removed leaving 50 sites, and richness was rarefied to the smallest sample size (either the live assemblage or the death assemblage). Sites (points) with high richness in the multi-taxic live assemblage (A–B) had correspondingly high richness in the death assemblage. When only molluscs were included (C–D), sites with a greater number of mollusc species in the live assemblage (LA) also had higher mollusc richness in the death assemblage (DA). Similarly, sites with high live assemblage non-mollusc richness had correspondingly high death assemblage non-mollusc richness (E–F), although this relationship was moderate, and not significant. Richness in mollusc death assemblages were also an excellent proxy for multi-taxic live assemblages (G–H), and sites with high richness in the multi-taxic live assemblage had correspondingly high richness in the mollusc death assemblage. The first column shows Spearman’s correlations (Rho), and the second column shows Pearson’s correlations (r). [file peerj-11-15574-s003.pdf]

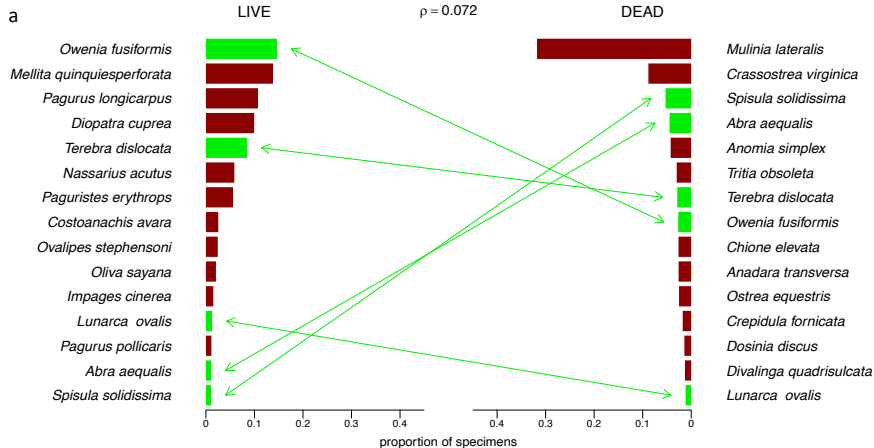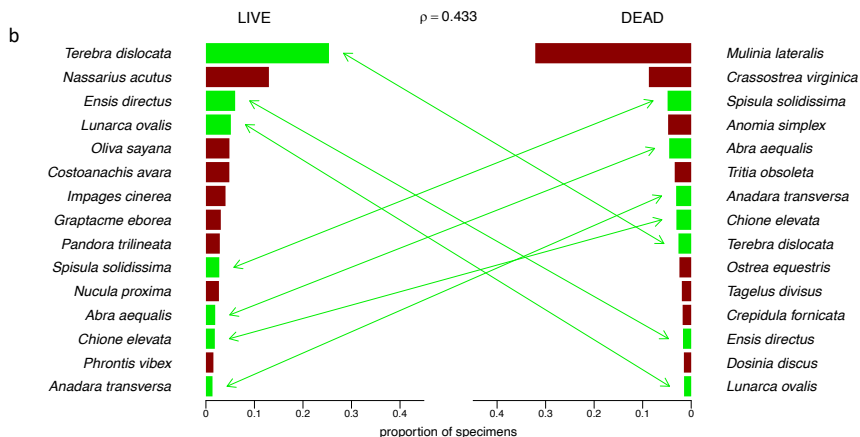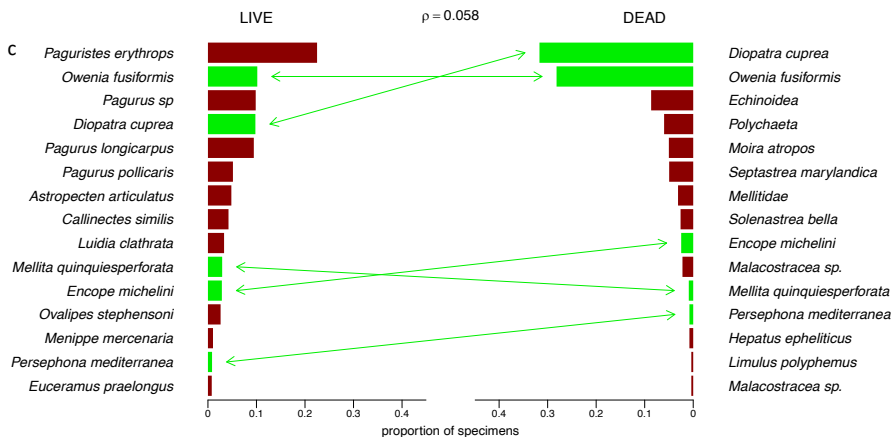

Supplement: Supplemental Information 4 — Taxa unique to either the LA or DA are shown in red, while taxa present in both the LA and DA are shown in green. Samples were standardized as above, and those with fewer than 20 specimens were removed. [file peerj-11-15574-s004.pdf]

rarefied number of species

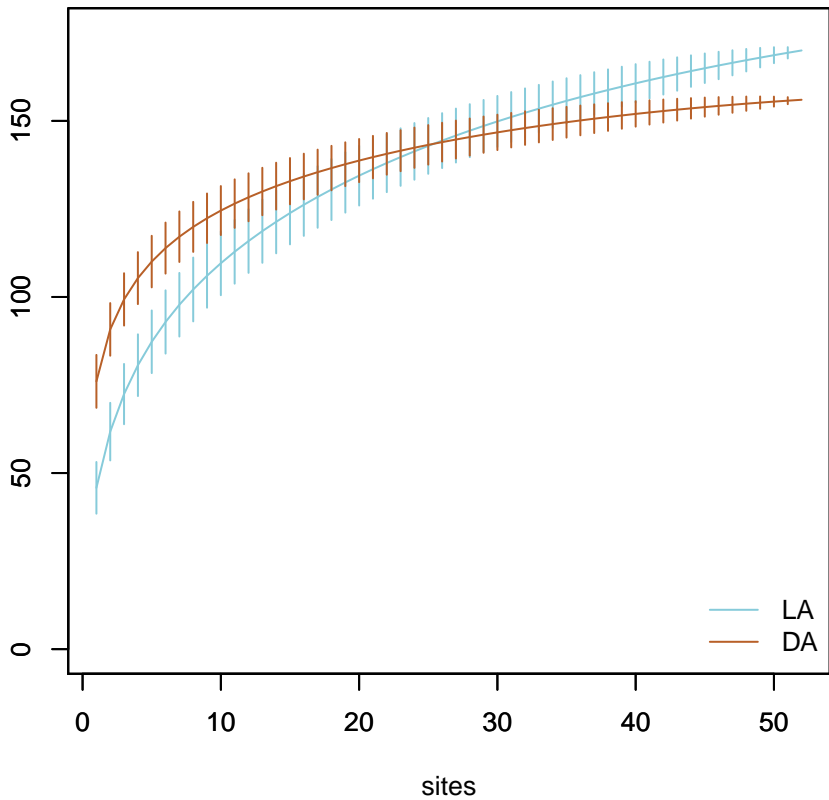

Supplement: Supplemental Information 5 — The accumulation of species for the full LA and DA (all 52 localities). Vertical bars represent the 95% confidence intervals. The curves were broadly congruent, with moderate offsets in the number of species between assemblages. Although the LA did not reach the asymptote, indicating moderate under-sampling, the slope suggests that the LA was nevertheless close to fully sampled and was approaching the asymptote. The slope of the DA suggests that the DA was somewhat more comprehensively sampled than the LA. Differences between the LA and DA are thus unlikely to be due to sampling incompleteness. [file peerj-11-15574-s005.pdf]

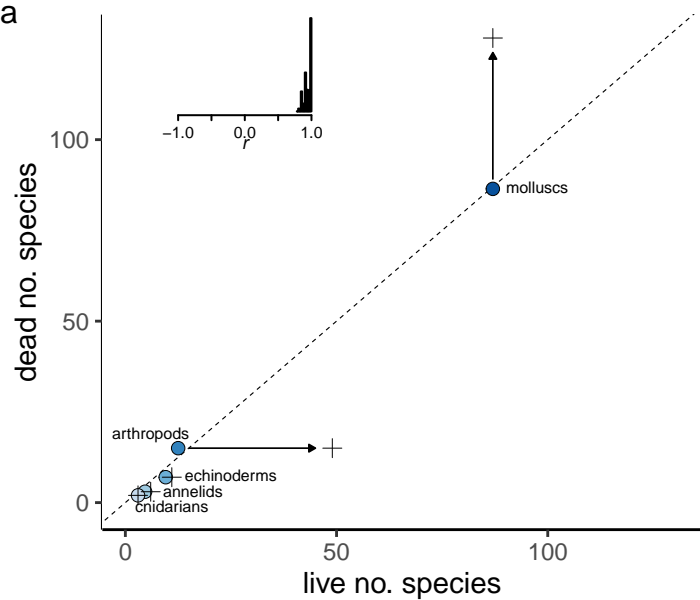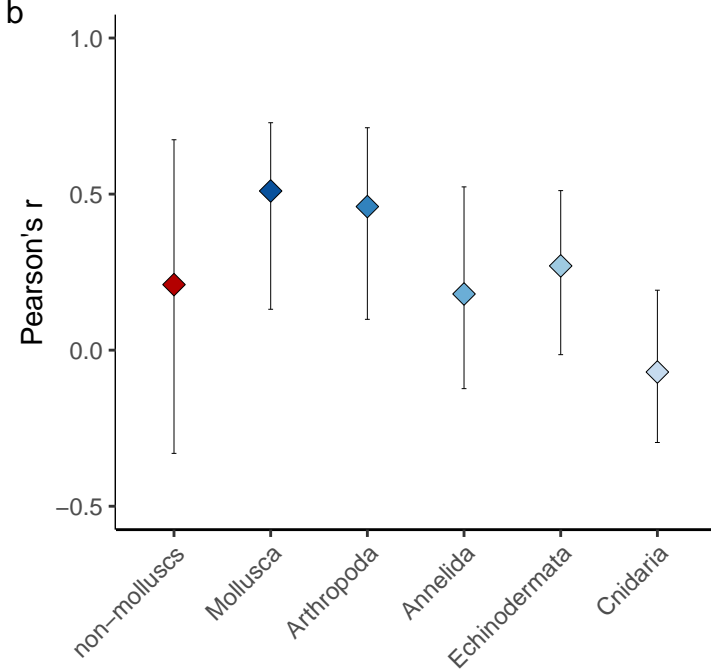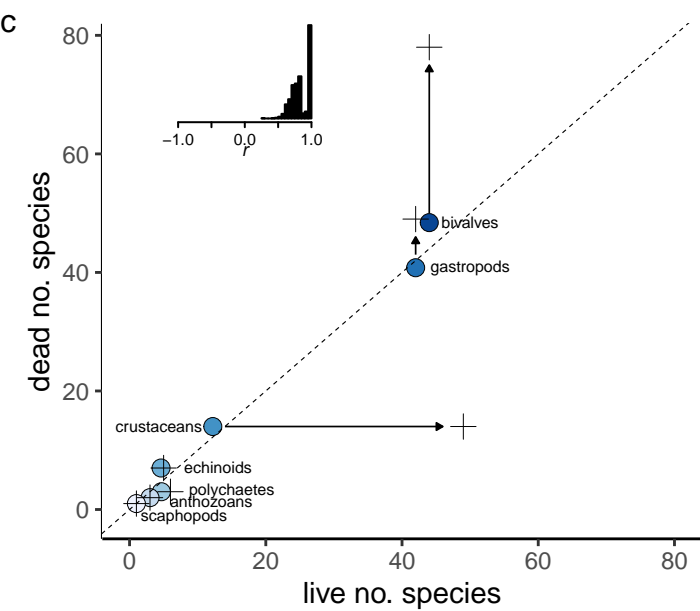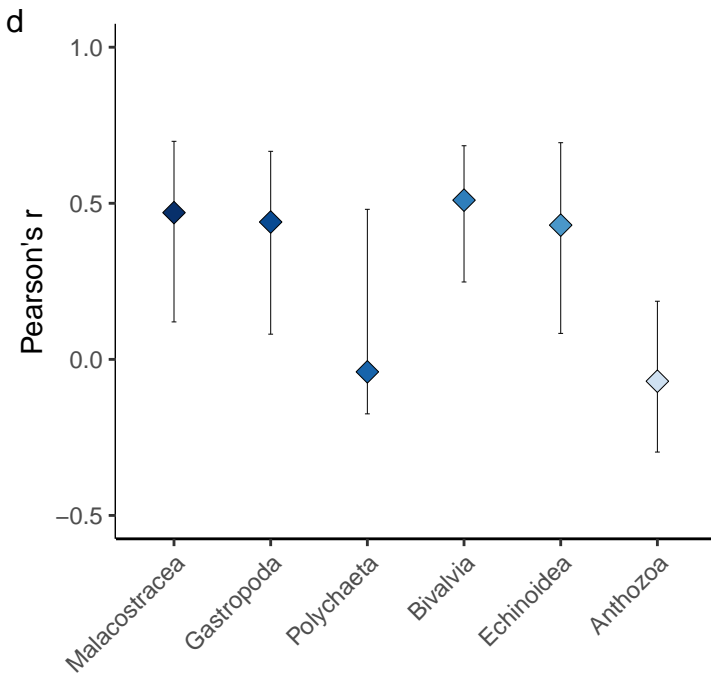

Supplement: Supplemental Information 6 — The number of species within phyla (A) and classes (C) indicate predictable live-dead discordance. Filled circles indicate live richness and crosses dead richness. Arrows show the change in richness between the LA and DA for higher taxa, and the dashed line denotes perfect fidelity. The fidelity of richness was also assessed within phyla (B) and classes (D) using Pearson’s correlations, with 95% confidence intervals calculated using an accelerated bootstrapped correction. Classes with fewer than three species were excluded. Samples with less than 20 individuals were removed, and samples were standardized. [file peerj-11-15574-s006.pdf]

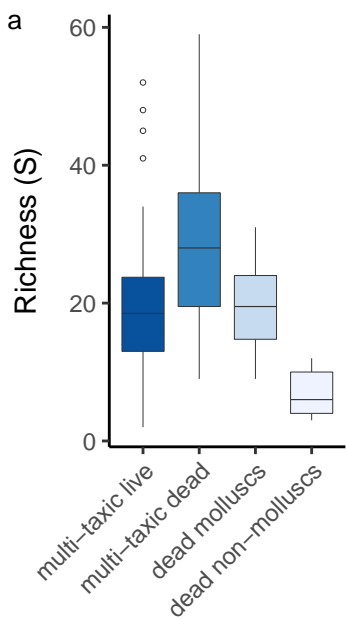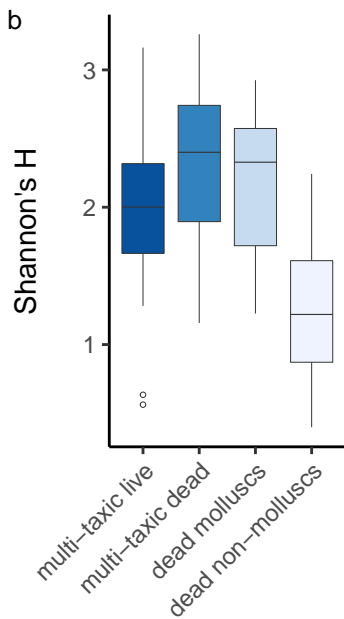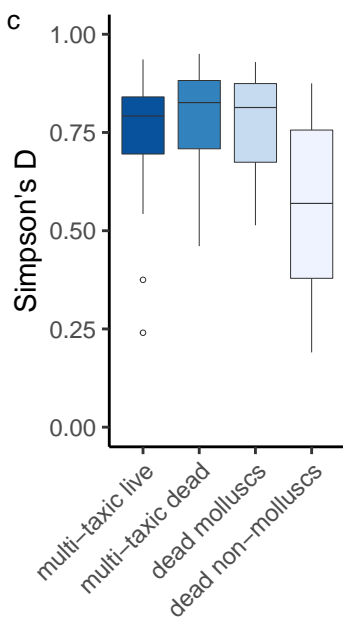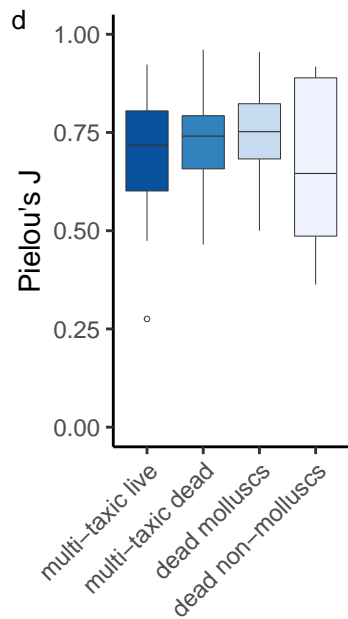

Supplement: Supplemental Information 7 — Sample standardized comparisons of Richness (S) (A), Shannon’s H (B), Simpson’s D (C), and Pielou’s J (D). For all measures of diversity, the mollusc DA does not differ significantly from the multi-taxic LA (Table S3). Mollusc death assemblages thus serve as reliable records of diversity in multi-taxic live assemblages. Samples with fewer than 20 individuals were removed. [file peerj-11-15574-s007.pdf]
